# Case of Congenital Deformity of Hands

**Published:** 1868-12

**Authors:** 


### Correspondence.

### Case of Congenital Deformity of Hands.

Big Flats, December 11th, 1868.

Dr. Julius F. Miner:

Dear Sir:—Having read your article in the *Buffalo Medical and Surgical Journal*, September, 1868, on the causes of "Congenital Club-foot," I send the following report of a case which may be thought to corroborate your view of the causes of some congenital deformities:

Mrs. Thomas Treat, aged 27, primipara, was delivered of a small but vigorous male infant, October 1st, 1868. The child was born before my arrival, but the circumstances indicated a small quantity of the liquor amnii, as the bed-clothes were not saturated; while the child was being dressed it was noticed that both hands were flexed horizontally upon the fore-arm, the wrists at the ulnar side were dislocated, and on the left hand there was not a vestige of a thumb; on the right hand a small thumb, with no natural articulation with the hand. By request of the parents it was amputated on the 5th instant, there being no prospect that it would be of any service. The hands were put in a natural position, and retained so by splints and bandages, and that treatment is now continued with apparent benefit. The absence of the thumb of one hand and the defective thumb on the other, may have been the result of the pressure against the fore-arms during intra-uterine life.

Yours, respectfully,

WM. WOODWARD, M. D.

The above report is received with much pleasure. In connection with the paper referred to, it is very opportune, and is illustrative of several important facts. Whoever reads them in connection will see the bearing it has upon the subject of the causes of congenital deformities. J. F. MINER.

